# Path-Tracking Control Strategy of Unmanned Vehicle Based on DDPG Algorithm

**DOI:** 10.3390/s22207881

**Published:** 2022-10-17

**Authors:** Jialing Yao, Zhen Ge

**Affiliations:** School of Automotive and Transportation Engineering, Nanjing Forestry University, Nanjing 210037, China

**Keywords:** unmanned vehicle, DCN-DDPG, path tracking, multiple working conditions, CARLA

## Abstract

This paper proposes a deep reinforcement learning (DRL)-based algorithm in the path-tracking controller of an unmanned vehicle to autonomously learn the path-tracking capability of the vehicle by interacting with the CARLA environment. To solve the problem of the high estimation of the Q-value of the DDPG algorithm and slow training speed, the controller adopts the deep deterministic policy gradient algorithm of the double critic network (DCN-DDPG), obtains the trained model through offline learning, and sends control commands to the unmanned vehicle to make the vehicle drive according to the determined route. This method aimed to address the problem of unmanned-vehicle path tracking. This paper proposes a Markov decision process model, including the design of state, action-and-reward value functions, and trained the control strategy in the CARLA simulator Town04 urban scene. The tracking task was completed under various working conditions, and its tracking effect was compared with the original DDPG algorithm, model predictive control (MPC), and pure pursuit. It was verified that the designed control strategy has good environmental adaptability, speed adaptability, and tracking performance.

## 1. Introduction

In recent years, autonomous driving has become the focus of development in the field of unmanned vehicles. Path tracking is the basis and core technology of autonomous driving. Therefore, unmanned vehicles must have a reliable path-tracking controller.

There are many traditional control methods of path tracking, which can be roughly divided into two categories: model-free control and model-based control. In model-free control, the system dynamics is regarded as a black box. The steering command is only generated based on the tracking error, such as proportional-integral-derivative control (PID). PID does not require the system to establish an accurate mathematical model to complete the tracking task. However, when the nonlinearity and uncertainty of the system are high, model-free control becomes unreliable because this method cannot accurately express the motion state of the system.

Model-based control includes kinematic model-based control and dynamic model-based control. Control based on the kinematics model usually simplifies the vehicle as a mass point. Control methods based on the kinematics model mainly include pure-pursuit control and Stanley control. The steering wheel angle of the vehicle obtained by this type of controller is usually related to the heading angle error and lateral error of the vehicle. This method is easy to implement, but it can only achieve the real-time response of the vehicle at low speeds, and is not suitable for tracking control in high-speed conditions [1,2]. When considering complex urban conditions and high-speed traffic environments, the reliability and robustness of the controller of the model are not highly based on kinematics, so a vehicle dynamics model needs to be introduced.

The control method based on the dynamic model considers multiple factors relating to the vehicle body and external interference items when the vehicle is running at high speeds; these factors include the nonlinear change in the tire, the yaw and roll constraints of the vehicle, the change in the road curvature, etc. The control method based on the dynamic model improves the safety and reliability of the vehicle in complex working conditions. The control methods based on the dynamic model mainly include the linear-quadratic regulator (LQR), H-infinity control, model predictive control (MPC), sliding mode control (SMC), etc. [3,4]. H-infinity control and sliding mode control can compensate for uncertainty and external disturbances in lateral control and are promising trajectory-tracking control techniques, but the main disadvantage of sliding mode control is chattering. The author in [5] introduced an adaptive mechanism together with a finite frequency H∞ control strategy to reject the effects of actuator faults and disturbances, respectively. The author in [6] proposed a method that combines the sliding mode approach with a smooth nonlinear control law to reduce amplitude, which limits the steering angle. In [7], high-order sliding mode control was used to reduce jitter. In [8], by establishing a speed-planning module, a steering control module, and a speed-tracking module, sliding mode control (SMC) with adaptive preview time was used to control the autonomous vehicle to track the target path. The author in [9] proposed a four-wheel PID control and SMC control based on the traditional two-wheel PID control and SMC control, which were used in automatic control vehicles with four-wheel independent steering and drive. Chattering will make the system oscillate or become unstable. All of the above optimal control methods can only reduce chattering, but not eliminate it. Eliminating chattering eliminates the anti-interference ability of sliding mode control, but the existence of chattering affects the stability of vehicle control and the comfort of vehicle riding. Due to the high computational complexity and poor real-time performance of the method based on the vehicle dynamics model, the vehicle dynamics model is usually a simplified two-degree-of-freedom bicycle model, which retains the yaw and lateral motion of the vehicle. On the basis of accurately describing the vehicle dynamics, the vehicle model is simplified as much as possible to reduce the calculation amount of the algorithm and ensure the real-time performance of the control system.

In addition to the above-mentioned traditional control methods, intelligent control methods are currently more favored by scholars. At present, the mainstream intelligent methods include fuzzy control, genetic algorithms, neural networks, reinforcement learning, etc. The author in [10] proposed a new control approach based on the immersion and invariance control theorem to ensure that the vehicle completes the vehicle path-tracking task under various operating conditions, parameter uncertainties, and external disturbances. Because intelligent algorithms are mainly model-free, many scholars have combined intelligent algorithms with traditional algorithms to solve the shortcomings of traditional methods. Some scholars proposed the combination of fuzzy control and PID [11]. Compared with traditional PID, fuzzy PID has better stability and robustness, and has better control accuracy, and can adapt to different road conditions. Some scholars combined the genetic algorithm and MPC [12], using the genetic algorithm to adjust parameters online, which made the controller less susceptible to external factors and improved tracking accuracy. Some scholars combined reinforcement learning and MPC [13]. This method adjusts the weight of MPC through reinforcement learning, which reduces the difficulty of adjusting the weight of MPC.

Deep reinforcement learning (DRL) is a kind of intelligent algorithm. It is a combination of deep learning and reinforcement learning. In the training process, deep reinforcement learning obtains the optimized goal through the interaction and exploration of reinforcement learning and the environment, and then uses deep learning to fit the control strategy. Deep reinforcement learning is mainly used to solve problems in the Markov decision process (MDP). It can obtain the optimal strategy for selecting actions in the environment through the agent, and the goal is to maximize the reward value. Compared with traditional control methods, such as H-infinity, which require fixed structures and parameters, deep reinforcement learning does not require complex models, so the amount of computation is relatively small. In the face of environmental changes, the rapid adjustment performance of deep reinforcement learning is superior to traditional control methods. Compared with other intelligent control methods, such as sliding mode control, reinforcement learning has higher accuracy and no chattering. At present, deep reinforcement learning has achieved considerable results in the field of games, and has also been widely studied in some fields that require complex control [14,15,16], such as smart ships, unmanned surface vehicles [17], and unmanned vehicles [18].

The author in [19] proposed a path-following control method for AUVs based on adaptive-reinforcement learning. Based on the continuous interaction with nonstationary environments, this method achieved the adaptive control of AUVs and obtained good simulation test results. However, this work only conducted a preliminary study on the algorithm. Only a set of simulation tests was performed, and the results were imperfect. The author in [20] proposed an MPQ-DPG algorithm to investigate trajectory tracking problems for a class of autonomous underwater vehicles (AUVs). The algorithm could achieve high-level tracking-control accuracy of AUVs and stable learning by applying a hybrid actors–critics architecture, where multiple actors and critics were trained to learn a deterministic policy and action-value function, respectively. The effectiveness and generality of the MPQ-DPG algorithm were verified by its application on an AUV with two different reference trajectories. However, the random experience extraction method in the algorithm made the agent fall into the local optimal. The author in [21] proposed double-critic networks and the priority experience replay deep deterministic policy gradient (DCPER-DDPG) algorithm to improve sample utilization and training speed. However, the convergence of the algorithm was not stable enough. The author in [22] designed a double experience replay buffer (DERB) to increase learning efficiency and accelerate convergence speed. However, the simulation scenario was simple and could not meet the actual requirements.

Because each step in the car’s driving is continuous, which is the same as the continuous characteristic of the deep deterministic policy gradient (DDPG), this paper proposes a path-tracking control strategy for unmanned vehicles based on the DCN-DDPG algorithm. The goal of the control strategy was for the unmanned vehicle to follow a predetermined route without collision in urban road environments. To verify the effectiveness of the control strategy, the proposed control strategy was simulated and tested in the CARLA simulator. CARLA is an open-source simulator that can simulate real traffic environments, pedestrian behaviors, car sensors, etc., while providing a dynamic urban environment. In CARLA, the current position of the reference vehicle can be obtained through a global navigation satellite system (GNSS). The unmanned vehicle path-tracking control strategy proposed in this paper introduces the change rate of the vehicle-heading angle as a constraint variable, which reduces the lateral jitter of the vehicle during the tracking process and ensures the stability of the vehicle tracking. In addition, the design of the reward value function solves the problem of deep reinforcement learning’s sparse reward value, causing difficulty in convergence. Finally, the tracking effect of the proposed path-tracking control strategy for unmanned vehicles based on the DCN-DDPG algorithm was verified in Town04, and compared with the original DDPG, MPC, and pure pursuit.

## 2. Related Models and Network Design

### 2.1. Path-Tracking Model

**Figure 1:**
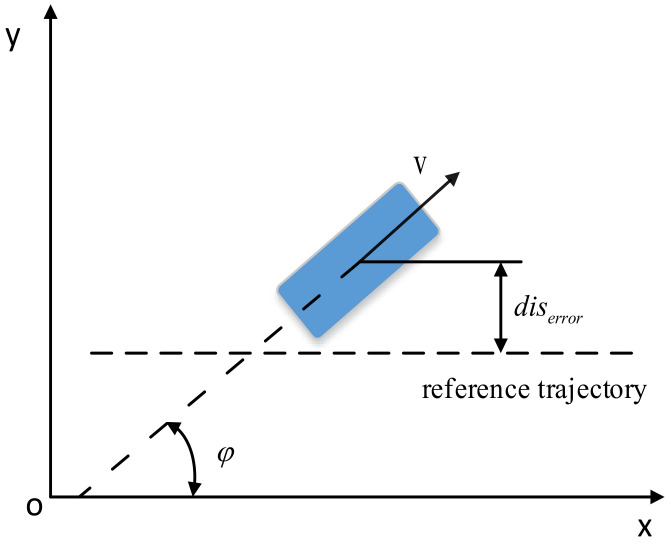
Vehicle tracking model.

Figure 1 shows the vehicle tracking model. In the geodetic coordinate system OXY, *v* is the speed of the vehicle, *φ* is the heading angle of the vehicle, and *dis_error_* is the lateral error of the vehicle from the reference trajectory.

### 2.2. Path-Tracking Controller Design

The essence of reinforcement learning (RL) is to continuously improve performance through interaction with the environment. The learners and decision makers in reinforcement learning are called agents, and the rest of the parts that interact with the agents are called the environment. DDPG is a model-free algorithm based on deterministic policy gradients, which is based on the actor–critic framework and applied in continuous action spaces.

#### 2.2.1. Overall Scheme Design

**Figure 2:**
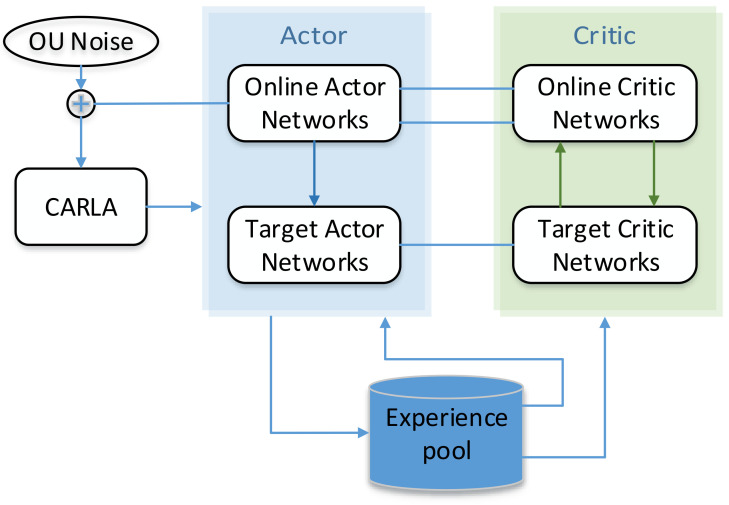
DDPG overall framework.

The path-tracking controller based on the DDPG algorithm includes four networks, an OU noise module, a reward value calculation module, and an experience pool. The four networks are the actor, critic, target actor, and target critic networks. The role of the OU noise module is to increase the exploration of strategies. The reward value calculation module can calculate the reward value for network learning according to the current state. The design of the experience pool can improve the utilization of data, and the overall framework is shown in Figure 2.

The input of the path-tracking controller based on the DDPG algorithm is $S_{t}$, which represents the state of the environment; $S_{t}$ is $\left[dis_{error},\varphi_{error},\dot{\varphi }\right]$, which is a state space composed of the lateral error between the unmanned vehicle and the reference path, the heading angle deviation, and the rate of change of the heading angle after normalization, respectively. $A_{t}$ is the steering wheel angle of the vehicle, which is determined by the input state quantity and control decision, thereby controlling the lateral movement of the vehicle. When the vehicle moves, the position and state of the vehicle accordingly change, so as to obtain $S_{t+1}$, the state at the next moment, and $R_{t}$, the reward value.

Before training, the environment, state space, action space, and network parameters that include $\theta^{Q}$, $\theta^{\mu }$, and $\theta^{\mu \prime }$ must be initialized. $S_{t}$ can be obtained through CARLA, and using $S_{t}$ as the input of the actor network can obtain $\mu (A_{t})$, the output of the actor network. After adding OU to $\mu (A_{t})$, $A_{t}$ can be obtained. Specifically, this is shown in Equation (1). After $A_{t}$ is transmitted to the unmanned vehicle, the vehicle enters $S_{t+1}$, and the $R_{t}$ that represents the reward value is accordingly generated. After each process, the sample $\left(S_{t},A_{t},R_{t},S_{t+1}\right)$ is stored in the experience pool.

(1) $$A_{t}=\mu (S_{t})+N_{t}$$

When the number of samples in the experience pool reaches a certain number, *N* samples are randomly selected for training. Then, calculating the current target value is shown in Equation (2).

(2) $$y_{t}=R_{t+1}+\gamma Q^{\prime }(S_{t+1},\mu^{\prime }(S_{t+1}|\theta^{\mu^{\prime }})|\theta^{Q^{\prime }})$$

The update of the critic network is performed by minimizing the loss L. The calculation process of L is shown in Equation (3).

(3) $$L=\frac{1}{n}{\sum_{t}\left(y_{t}-Q(S_{t},A_{t}|\theta^{Q})\right)}^{2}$$

The update of the actor network parameters, $\theta^{Q}$ and $\theta^{\mu }$, is performed by gradient descent. The update of the critic network parameters, $\theta^{Q\prime }$ and $\theta^{\mu \prime }$, is performed by softupdate every 3 steps. The update process of critic network parameters is shown in Equation (4).

(4) $$\left\{\begin{array}{l} \theta^{Q\prime }\leftarrow \tau \theta^{Q}+(1-\tau )\theta^{Q\prime }\\ \theta^{\mu \prime }\leftarrow \tau \theta^{\mu }+(1-\tau )\theta^{\mu \prime } \end{array}\right.$$

When the vehicle collides or the lateral error reaches the threshold, the program exits the current loop and enters the next loop. The actor and critic networks in the controller are both composed of fully connected layers, and the optimizer is the Adam optimizer. The structures of the actor network and the critic network are shown in Table 1 and Table 2, respectively.

#### 2.2.2. Design of Reward Value Calculation Module of DDPG Controller

The reward-value design of reinforcement learning is generally ‘0–1′, which means the reward value for reaching the target is 1, and the reward value for failing to achieve the target is 0. This method is likely to cause the reward value to be sparse and lead to low learning efficiency. In order to solve the problem of sparse reward value, the reward value calculation module was redesigned, as shown in Equation (5).

(5) $$R_{t}=0.6\times R_{1}+0.3\times R_{2}+0.1\times R_{3}$$

**Figure 3:**
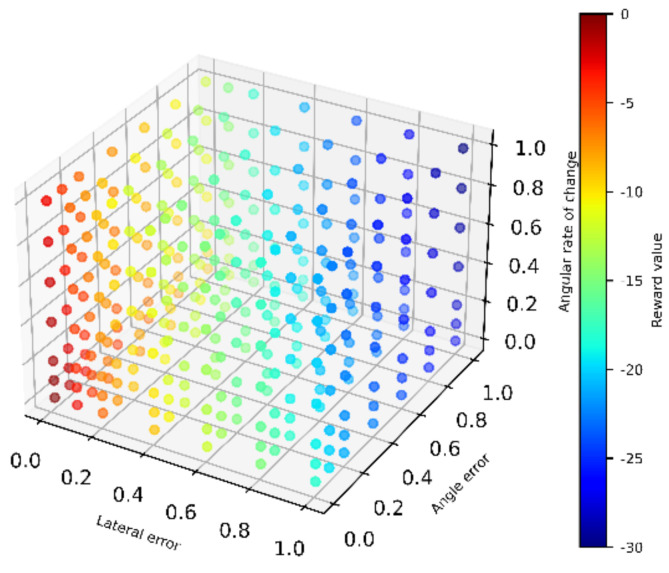
DDPG overall framework.

In the formula, $R_{t}$ is the total reward value; $R_{1}$, $R_{2}$, and $R_{3}$ are the reward values for distance error, heading error, and rate of change of heading, respectively; $R_{1}$, $R_{2}$, and $R_{3}$ are shown in Equations (6)–(8), respectively. The relationship between the total reward value and $R_{1}$, $R_{2}$, and $R_{3}$ is shown in Figure 3.

(6) $$R_{1}=\left\{\begin{array}{cc} 0 & dis_{error}\leq 0.01\\ -5\times ceil\left(\frac{abs(dis_{error})}{0.1}\right) & 0.01<dis_{error}\leq 0.2\\ -5-5\times ceil\left(\frac{abs(dis_{error})}{0.2}\right) & 0.2<dis_{error}\leq 1 \end{array}\right.$$

(7) $$R_{2}=\left\{\begin{array}{cc} 0 & \varphi_{error}\leq 0.01\\ -5\times ceil\left(\frac{abs(\varphi_{error})}{0.1}\right) & 0.01<\varphi_{error}\leq 0.2\\ -5-5\times ceil\left(\frac{abs(\varphi_{error})}{0.2}\right) & 0.2<\varphi_{error}\leq 1 \end{array}\right.$$

(8) $$R_{3}=\left\{\begin{array}{cc} 0 & \dot{\varphi }\leq 0.01\\ -5\times ceil\left(\frac{abs(\dot{\varphi })}{0.1}\right) & 0.01<\dot{\varphi }\leq 0.2\\ -5-5\times ceil\left(\frac{abs(\dot{\varphi })}{0.2}\right) & 0.2<\dot{\varphi }\leq 1 \end{array}\right.$$

#### 2.2.3. Double Critic Networks of DDPG Algorithm

To solve the problems of the overestimation of the Q-value, large accumulated error, and slow training speed of the DDPG algorithm, this paper adopted the following steps to design the DCN-DDPG algorithm:

The target critic network consists of two critic networks, CN1 and CN2.

(9) $$y_{t1}=R_{t+1}+\gamma Q_{1}{}^{\prime }(S_{t+1},\mu_{1}{}^{\prime }(S_{t+1}|\theta^{\mu^{\prime }})|\theta^{Q_{1}{}^{\prime }})$$

(10) $$y_{t2}=R_{t+1}+\gamma Q_{2}{}^{\prime }(S_{t+1},\mu_{2}{}^{\prime }(S_{t+1}|\theta^{\mu_{2}{}^{\prime }})|\theta^{Q_{2}{}^{\prime }})$$

where $R_{t+1}$ is the current reward value, γ is the discount factor, and $Q_{1}{}^{\prime }$ and $Q_{2}{}^{\prime }$ are the values calculated by two target critic networks, respectively.

(11) $$y_{t}=R_{t+1}+\gamma \times \left|\frac{\underset{i=1,2}{\min}Q_{i}{}^{\prime }}{\underset{i=1,2}{\max}Q_{i}{}^{\prime }}\right|\times \underset{i=1,2}{\min}Q_{i}{}^{\prime }$$

## 3. Results

### 3.1. Simulation Environment and Parameter Configuration

The CPU model of the simulation hardware used in this study was i5-12400F (Intel+Asus, Shanghai, China); the GPU model was RTX3060 (Colorful, Shenzhen, China); the memory stick had a capacity of 16G (CRUCIAL, Meridian, America); and the operating system was Windows 10. The simulation software environment was jointly built by CARLA and Python. The main parameters of DCN-DDPG were set as shown in Table 3.

In order to verify the tracking effect of the DCN-DDPG controller under different working conditions, the experimental environments of this paper were divided into three categories, as shown in Table 4.

**Table 4:** Experimental environment.

Experimental Environment Name	Environmental Description	Experimental Purpose	Example Diagram
Linear tracking	The reference path is a straight line with no turns	Validate the tracking ability of the vehicle on a straight road	Figure 4a
Ramp tracking	The reference path is a ramp with consecutive turns	Validate the vehicle’s tracking ability in continuous curves	Figure 4b
Complex environment tracking	Reference paths are complex road segments, including turns and straight lines	Validate the tracking ability of vehicles in complex road sections	Figure 4c

### 3.2. Analysis of DCN-DDPG Training Results

The two curves in Figure 5 show the average number of steps and the average reward value of the DCN-DDPG algorithm in each iteration process. It can be seen from Figure 5 that the number of steps and the reward value were small at the initial stage of training and had a certain randomness. In the middle stage of training, the average number of steps significantly increased, indicating that the number of vehicle collisions gradually decreased, and the tracking effect was improving. In the later stage of training, both the average number of steps and the average reward value showed a convergence trend, which verified the effectiveness of the algorithm.

The two curves in Figure 6 show the average reward values of the DDPG and DCN-DDPG algorithms. The DDPG algorithm did not converge until the 450th iteration, while the DCN-DDPG algorithm converged at only the 300th iteration. For the entire 1700 training sessions, the training speed was increased by 8.82%.

### 3.3. Environmental Suitability Verification

**Figure 4:**
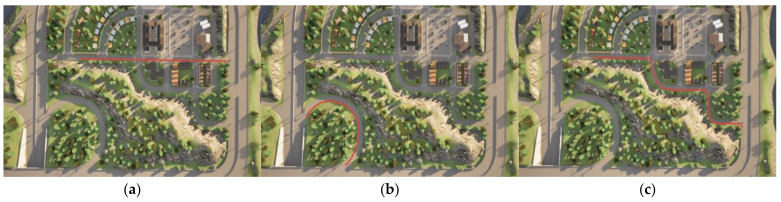
Experimental environment diagram: (**a**) linear tracking environment, (**b**) ramp tracking environment, and (**c**) complex tracking environment.

**Figure 5:**
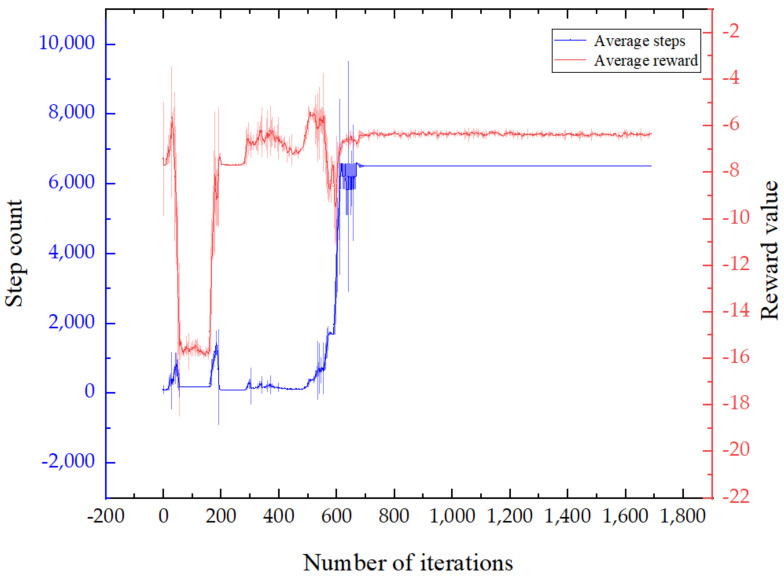
Training results.

**Figure 6:**
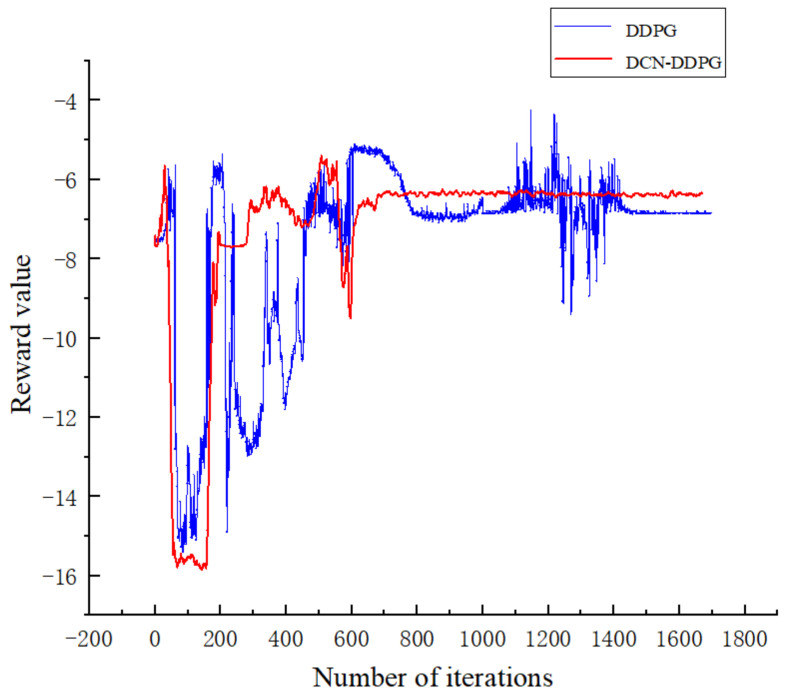
Comparison of training result graphs.

In order to test the tracking effect of the DCN-DDPG lateral controller in different scenarios, the Model 3 model in CARLA was selected and the tracking task was performed on the three roads in Figure 4. The tracking results are shown in Figure 6.

**Figure 7:**
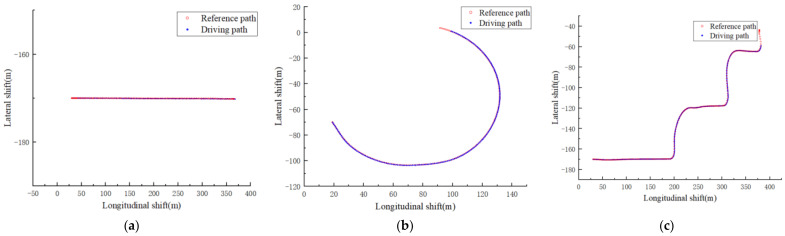
Tracking results in different environments: (**a**) line-tracking global map, (**b**) global map of ramp tracking, and (**c**) global map of complex path tracking.

Figure 7a–c show the tracking effect of Model3 on straight lines, ramps, and complex paths, respectively. It can be seen that the path-tracking controller based on the DCN-DDPG algorithm designed in this paper could make unmanned vehicles complete tracking tasks in different environments.

### 3.4. Comparative Analysis of Tracking Effects of Different Methods

**Figure 8:**
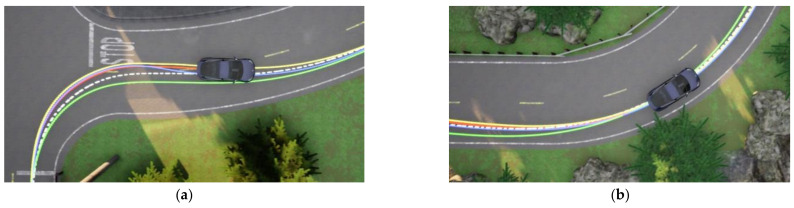
Tracking comparison of different control methods: (**a**) comparison of the first corner and (**b**) comparison of the last corner (the white line in Figure 8 represents the reference path; the green line represents the driving route of the pure pursuit method; the blue line represents the driving route of the MPC method; the yellow line represents the driving route of the DDPG method; the orange line represents the driving route of the DCN-DDPG method designed in this paper).

On complicated roads, the unmanned vehicle tracked the reference path with three different methods: DDPG, DCN-DDPG, MPC, and pure pursuit. When Model 3 passed through the curve, the unmanned vehicle entered a short-term deviation-adjustment state, and changed from a large error to a small error (0.07 m). The enlarged partial view of the lateral deviation generated by the three methods is shown in Figure 8. Figure 8a shows the comparison of the tracking effect of the unmanned vehicle in the first curve in the environment of Figure 4c with different control methods, and Figure 8b shows the comparison of the tracking effect of the unmanned vehicle in the last curve in the environment of Figure 4c with different control methods.

In CARLA, the input range of the steering wheel was [−1, 1]. During the tracking process of the three methods of DDPG, DCN-DDPG, MPC, and pure pursuit, the maximum value of the steering wheel angle ratio was 0.81, 0.98, 0.94, and 0.96, respectively. The average value of the steering wheel angle ratio was 0.11, 0.16, 0.12, and 0.14, respectively, in which MPC had the best effect. The maximum values of absolute values of lateral errors of Model 3 controlled by these three methods were 0.66, 0.63, 0.73, and 0.95 m, respectively. The average values of absolute values of lateral errors were 0.14, 0.08, 0.13, and 0.52 m, respectively. The tracking effect of DCN-DDPG was superior. The response durations of the DDPG, DCN-DDPG, and MPC deviation adjustment states at the first corner were 2.93, 2.27, and 2.31 s, respectively, and pure pursuit could not enter the deviation adjustment state due to the large deviation. Among them, the deviation adjustment time of DCN-DDPG was the shortest. The following conclusions could be drawn: in the face of emergencies, the unmanned vehicle with DCN-DDPG as the controller could make adjustments in a short time, and the tracking effect is shown in Table 5.

### 3.5. Speed Suitability Verification

**Figure 9:**
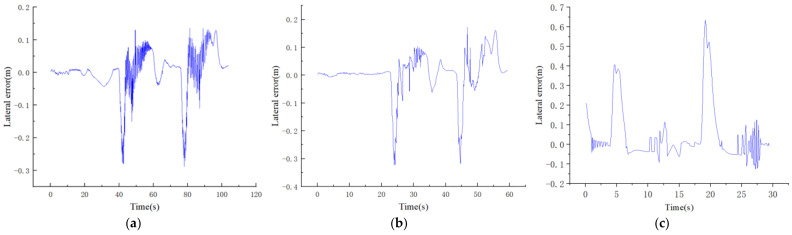
Lateral error of tracking at (**a**) 4 m/s, (**b**) m/s, and (**c**) 10 m/s.

In order to verify the adaptability of the path-tracking controller designed in this paper with different speeds, the unmanned vehicle was tracked in the environment shown in Figure 4c at speeds of 4, 7, and 10 m/s, and the test results are shown in Figure 9.

It can be seen from Figure 8 that during the trajectory tracking process of the vehicle, as the vehicle speed increased, the absolute value of the maximum lateral error value also increased, and the absolute values of the maximum lateral error values during tracking at speeds of 4, 7, and 10 were 0.3, 0.36, and 0.63 m, respectively. All of them occurred at sharp turns, and the lateral errors generated by other working conditions were small, which met the requirements of vehicle trajectory tracking.

## 4. Conclusions

(1) This paper utilized an unmanned vehicle as the research object and used the deep-reinforcement learning algorithm to study the optimal control problem of the vehicle when tracking the path. The vehicle-tracking control strategy was learned with two mutually updated neural networks, critic and actor, and the DDPG framework was used to update the parameters of the neural network. A new reward value function was designed, which took into account the rate of change of the heading angle to reduce lateral shaking of the vehicle.
(2) This paper designed a DCN-DDPG algorithm. Based on the original DDPG algorithm, a double-critic network was designed to solve the problems of high Q-value and slow training speed.
(3) In the CARLA simulator, the designed control strategy was applied to different scenarios, and the vehicle ran at different speeds to test the adaptability of the proposed tracking control strategy to the speed. The results showed that the proposed path-tracking strategy has a good tracking effect. The vehicle could complete the path-tracking task at different speeds and had good adaptability to the environment in which the path was tracked.
(4) Compared with the tracking effects of original DDPG, MPC, and pure pursuit methods, it was verified that the path-tracking controller designed in this paper can meet the conditions of vehicle path tracking.

## Figures and Tables

**Table 1:** Actor network structure.

Name	Actor Network		
	Input Dimension	Output Dimension	Activation Function
Input layer	3	50	Relu
Hidden layer	50	30	Relu
Output layer	30	1	Tanh

**Table 2:** Critic network structure.

Name	Critic Network		
	Input Dimension	Output Dimension	Activation Function
Input layer	4	60	Relu
Hidden layer	60	10	Relu
Output layer	10	1	none

**Table 3:** Main parameters of DCN-DDPG.

Parameter	Value
Sampling time (s)	10^−3^
Critic network initial learning rate	5 × 10^−3^
Actor network initial learning rate	3 × 10^−4^
Update parameters τ	10^−3^
Discount factor γ	0.99
Batch size	64
L2 regularization factor λ	6 × 10^−3^
Number of iterations	1700

**Table 5:** Path-tracking results comparison.

Parameter	Path-Tracking Results for Different Methods			
	DDPG	DCN-DDPG	MPC	Pure Pursuit
Maximum value of steering wheel angle ratio	0.81	0.98	0.94	0.96
Average value of steering wheel angle ratio	0.11	0.16	0.12	0.14
The maximum value of the absolute value of the lateral deviation/m	0.66	0.63	0.73	0.95
The mean value of the absolute value of the lateral deviation/m	0.14	0.08	0.13	0.52
Response time for bias adjustment status/s	2.93	2.27	2.31	

## Data Availability

Not applicable.
